# Dopaminergic and Noradrenergic Contributions to Divergent and Convergent Creativity Task Performance, a Systematic Review

**DOI:** 10.3390/bs15091185

**Published:** 2025-08-30

**Authors:** David Q. Beversdorf

**Affiliations:** Department of Neurology, Radiology, and Psychological Science, William and Nancy Thompson Endowed Chair in Radiology, University of Missouri, Columbia, MO 65211, USA; beversdorfd@health.missouri.edu

**Keywords:** creativity, dopamine, norepinephrine, stress, Parkinson’s, Tourette’s, attention deficit hyperactivity disorder

## Abstract

A range of factors affect performance on tasks associated with creativity, including stress, pharmacology, behavioral interventions, and neural stimulation strategies. Most of the pharmacological impacts have focused on the dopaminergic and noradrenergic systems. However, some evidence suggests that these systems may differ in their impact on performance on divergent and convergent tasks. We performed a systematic PubMed review examining the results for ‘creativity and (dopamine or dopaminergic)’ and ‘creativity and (norepinephrine or noradrenergic or adrenergic)’. The dopaminergic search yielded 238 articles. The noradrenergic search yielded 102 articles. The articles were screened for actually targeting these systems (for example, by pharmacological intervention or by examining genetics relevant to these systems) and examining performance on divergent or convergent tasks. Results of the review of the qualifying articles lent some support to an impact of the dopaminergic system on divergent task performance, and suggested an impact of the noradrenergic system on convergent task performance, but often required additional factors such as stress to reveal robust effects. This review suggests a differential impact of the dopaminergic system and noradrenergic system on divergent and convergent task performance, but studies to systematically address this question across conditions are lacking, and would be needed going forward.

## 1. Introduction

A range of factors have been identified that influence performance on tasks associated with creativity. Creativity performance is known to be impacted by psychiatric conditions (Andreasen, 2008; Power et al., 2015). Additionally, stress has been shown to impact performance on creativity-associated tasks (Beversdorf, 2020; Martindale & Greenough, 1975). This has led to a particular interest in the dopaminergic and noradrenergic systems for their impact on creativity (Beversdorf, 2020). Past research has demonstrated that performance on the alternate uses task is related to eyeblink rate, a marker associated with dopaminergic activity (Chermahini & Hommel, 2010), and additionally, D2 receptor polymorphisms predict performance on tasks such as object use fluency (Reuter et al., 2006). The noradrenergic system is most widely recognized for its role in arousal (Coull et al., 2004; Smith & Nutt, 1996). Drugs that block the beta-adrenergic receptors have been shown to reverse the effects of test anxiety (Faigel, 1991), an effect also observed for performance on anagram tasks (Alexander et al., 2007). However, there are two broad categories of creativity-associated tasks utilized in these exemplars. Tasks such as the alternate uses task and object use fluency involve a search to generate multiple potential ‘creative’ responses (divergent tasks). This requires the individual to come up with an open-ended set of responses to a query, like how many uses can you think of for a brick, and the novelty (often rated for novelty by blinded raters), fluency (how many responses are generated), and number of different categories covered amongst the responses are all potential outcomes that can be monitored to assess ‘creativity’ as it relates to divergence. For tasks such as the anagrams task, the unconstrained search converges onto one correct answer (convergent tasks) (Beversdorf, 2020; Heilman et al., 2003). This requires the individual to look at a problem, such as unscrambling sets of letters to form a word (anagrams—the solution for ‘dhilnop’ is ‘dolphin’), finding a word that forms a compound with three presented cue words (Compound Remote Associates—The solution for ‘apple, cone, tree’ is ‘pine’), or rearranging matchsticks to form a novel figure. These problems, as assessed in creativity research, involve a search through a broad network of potential solutions in order to converge on one correct answer, thus assessing ‘creativity’ as it relates to convergence. Problems that do not engage any such broad search of potential solutions, but rather simply iterative processing to derive the solution, would not be appropriate tasks in this setting as no creativity is required. To the author’s knowledge, there has never been a systematic effort to determine how the dopaminergic and noradrenergic systems might differentially impact performance on divergent and convergent tasks. To begin to address this, we performed a systematic PubMed review to examine the extant literature regarding the effects of the dopaminergic and noradrenergic systems on divergent and convergent task performance.

## 2. Methods

We performed a PubMed review examining the results for ‘creativity and (dopamine or dopaminergic’ and for ‘creativity and (norepinephrine or noradrenergic or adrenergic)’, from the entire PubMed database up until 24 March 2024 (INPLASY202570102). This search revealed 238 articles for the dopaminergic system and 102 for the noradrenergic system. The titles and abstracts were then screened for appropriateness. For the dopaminergic system, 106, and for the noradrenergic system, 68 were excluded as creativity was only mentioned, including cases where it appeared as describing their ‘creative approach’, and was not the focus of the research. Articles that did not produce new data, including review articles and commentaries, resulted in the additional exclusion of 64 articles for the dopaminergic system, and 20 for the noradrenergic system. There were an additional 22 dopaminergic system articles and 2 noradrenergic system articles excluded as they were not based on a task that could be utilized for the contrast between divergent and convergent creativity tasks. One additional paper was excluded for the dopaminergic system as it was exclusively a modeling paper, and four dopaminergic system articles and two noradrenergic system articles were excluded as they examined drugs impacting too broad a set of neuropharmacological systems to assess specific impact, and two additional dopaminergic system articles were excluded because they did discuss creativity and did discuss dopaminergic drugs, but not the effect of dopaminergic drugs on tasks related to creativity. The resulting set included 39 articles for the dopaminergic system, and 10 articles for the noradrenergic system (see Supplemental Figure S1 for summary). There were several articles that were relevant to BOTH the dopaminergic system and the noradrenergic system, and additionally, 3 of the dopaminergic papers were moved to the BOTH category as they involved genes that impact both the dopaminergic and noradrenergic systems (catechol-O-methyl transferase (COMT)), so the final categorization was 33 articles for the dopaminergic system, 7 articles for the noradrenergic system, and 7 articles for the category of both the dopaminergic and noradrenergic systems. Additionally, 12 articles known to the author were added to the noradrenergic system category that were not captured by the search strategy, as some of the earlier literature used the term ‘cognitive flexibility’ rather than ‘creativity’ for this particular topic. Furthermore, eight articles known to the author were added to the BOTH category, as the pharmacological systems were not always revealed by the search strategy for some studies on the effects of stimulants.

## 3. Results

**Figure 1:**
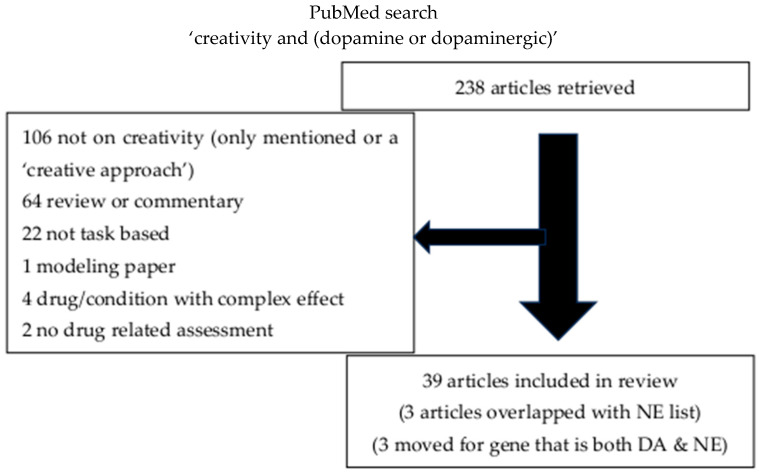
Articles retrieved on updated search (3/24/24) included and reasons for article exclusion for dopamine.

**Figure 2:**
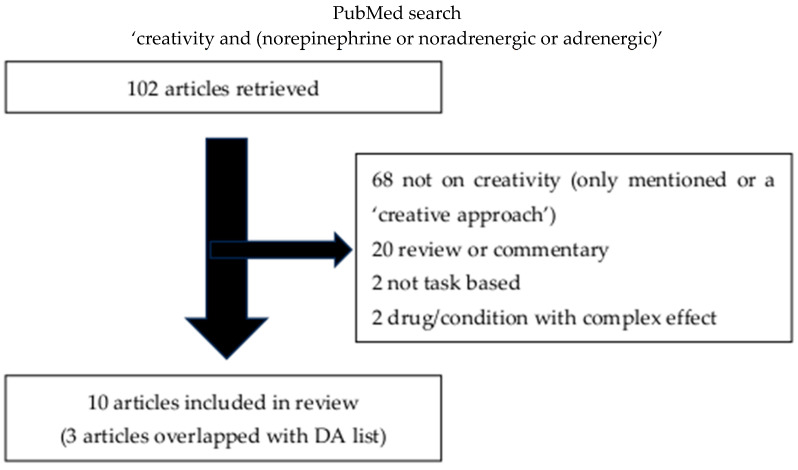
Articles retrieved on updated search (3/24/24) included and reasons for article exclusion for norepinephrine.

The remaining articles were studies examining the effect of pharmacological systems on either divergent or convergent tasks (Figure 1 and Figure 2). Results were tabulated according to which task type of assessment of the pharmacological system was examined for each pharmacological group. Within each table, articles were divided into 1—Studies with dopaminergic (DA) and/or noradrenergic (NE) drugs, 2—Studies with DA- and/or NE-associated diseases (DA only), 3—Studies with DA- and/or NE-associated diseases and drugs (DA only), 4—Studies with DA- and/or NA-related tasks, 5—Studies with DA and/or NE genes, 6—studies with DA and/or NE targeted by stress (NE only), 7—Studies with DA and/or NE targeted via sleep phase (NE only), 8—Studies with DA and/or NE imaging correlates (DA only), and 9—Studies with DA and/or NE targeted via electrical stimulation (Table 1, Table 2 and Table 3). Of note, the direction of the genetic studies as well as the imaging studies could have varying implications, so the direction of effect was not indicated in the table for these categories. For example, increased activation on imaging could relate to better performance, or could relate to greater effort required to perform the task, and decreased function of one specific receptor due to a genetic variant might result in compensatory increased activity of the system acting on other receptors for that neurotransmitter. Additionally, the COMT gene, which is usually reported as a dopaminergic gene, can impact both the dopaminergic and noradrenergic systems, so it was categorized under the grouping for both DA and NE (See Table 3).

Overall, it is readily apparent that the number of studies examining the effect of the DA system (33) exceeds the number of studies examining the effect of the NE system (21), even after inclusion of the added articles.

Of the articles examining the DA system, 29 examined the effect on divergent tasks, and for the directionality of the effects, 6 showed increased performance with increased DA activity (2 studies with dopaminergic tasks, 2 studies with dopaminergic diseases, 1 with dopaminergic diseases plus dopaminergic medications, and 1 with dopaminergic medications), 1 showed decreased performance (a study in Parkinson’s disease), 1 showed mixed effects (a cocaine study), and 3 showed no effect with increased DA (2 studies with dopaminergic diseases and 1 with dopaminergic diseases plus dopaminergic medications). Additionally, 10 examined the effect on convergent tasks. For those with clear directionality, two showed increased performance with increased DA activity (one study with dopaminergic tasks, one with dopaminergic diseases), three showed decreased performance (two studies with dopaminergic drugs, and one with dopaminergic tasks), and two showed no effect on performance with increased DA (one study with dopaminergic drugs, and one with dopaminergic diseases plus dopaminergic drugs) (Table 1).

Of the articles examining the NE system, only two examined the effect on divergent tasks, and for those with clear directionality, only one showed increased performance with NE antagonism (a study examining the relationship between performance and monitoring of sympathetic stress systems). All 21 of the articles examined the effect on convergent tasks, and for those with clear directionality, 17 showed increased performance with NE antagonism (8 noradrenergic drug studies, 6 stress studies, 1 stress plus noradrenergic drug study, 2 sleep phase studies, and 1 study examining body position effects), and 3 showed no effect (1 noradrenergic drug study, and 2 stress studies) (Table 2).

Of the 15 articles examining the impact of both the DA and NE systems, 14 examined effects on divergent tasks, and for those with clear directionality, 3 showed increased performance, 2 showed mixed performance, and 5 showed no effect. Additionally, 6 examined effects on convergent tasks, with 2 showing increased performance, and 2 showing no effect among those with clear directionality. Of note, all but one of these studies with clear directionality were drug studies with stimulants impacting both DA and NE systems, and 1 was with administration of a precursor, tyrosine, to both DA and NE (Colzato et al., 2015) (Table 3).

Of all of the 36 studies examining the impact of DA systems, only 6 examined effects on both divergent and convergent tasks. Administration of cocaine showed mixed effects on divergent tasks and decreased performance on convergent tasks (Hutten et al., 2019). Patients with Parkinson’s showed worse performance on divergent tasks (better performance with greater DA) but no effect on convergent tasks (Heldmann et al., 2024), and patients with Tourette’s showed no effect on divergent task performance but better convergent performance (Colautti et al., 2023). Administration of DA drugs in Parkinson’s patients showed no effect on either divergent or convergent performance (Salvi et al., 2021). Eyeblink rate was associated with better divergent task performance but worse performance on convergent tasks (Chermahini & Hommel, 2010), and fMRI activation in DA-associated region was found to be associated with performance on both divergent and convergent tasks (Aberg et al., 2017) (Table 1).

Among the 21 studies examining impact of NE systems, stress-related monitoring of the sympathetic system was associated with impact on divergent tasks, but not convergent tasks (Guo et al., 2024), and vagal nerve stimulation impacted performance on both divergent and convergent tasks (Ghacibeh et al., 2006). No other studies on NE systems examined effects on both divergent and convergent tasks (Table 2).

Among the 15 studies examining impact of BOTH systems, stimulants in ADHD patients improved divergent task performance with no effect on convergent tasks (McBride et al., 2021), or demonstrated no effect on either type of task (Boot et al., 2017), but when administered to individuals without ADHD, there was no effect on either type of task (Baas et al., 2020), or situational effects on convergent tasks only (Farah et al., 2009). Administration of the catecholaminergic precursor tyrosine, which is a precursor to both dopamine and norepinephrine, in healthy individuals increased convergent task performance without effect on divergent task performance (Colzato et al., 2015). No other studies on BOTH systems examined effects on both divergent and convergent tasks (Table 3), a gap in the literature noted in a recent review on this topic (Hoogman et al., 2020).

## 4. Discussion

This is, to our knowledge, the first effort to comprehensively review the literature on the relationships between the DA and NE systems and creativity. The current literature is rather limited in its ability to definitively determine selectivity of effects of the DA and NE systems on divergent and convergent task performance.

Overall, there is a tendency for the DA system to impact performance on divergent tasks, with increased DA associated with better performance. The literature on the DA system and convergent tasks is quite limited. Systematic studies examining the effect of the DA system on both divergent and convergent tasks are needed. An effect of DA on divergent task performance is also of interest given evidence that the DA system can impact a range of generative tasks not typically associated with creativity, as supported by its impact on motivational behaviors (Salamone & Correa, 2012), as well as persistence and effort (Walton & Bouret, 2019). Future studies will need to disentangle these effects for specificity to creativity.

There is also a tendency for the NE system to impact performance on convergent tasks, with decreased NE associated with better performance, and the literature on divergent tasks is limited. Systematic studies are also needed examining the effect of the NE system on both divergent and convergent tasks. Of further note, many of the studies demonstrating effects of the NE system on convergent task performance required the presence of an additional condition increasing the NE activity, alongside medications to decrease NE activity, in order to maximize the contrast, such as the induction of stress reversed by the effect of propranolol, a beta-adrenergic antagonist (Alexander et al., 2007) This methodological challenge will need to be considered in future explorations in this regard.

Regarding the studies examining the effect of stimulants, subsequent work demonstrated that autonomic arousal as assessed by heart rate variability did not predict the effect of stimulants on creativity task performance (Appling et al., 2025), suggesting that the effects of stimulants on divergent task performance from the same dataset in ADHD patients (McBride et al., 2021) were not mediated by NE effects.

The effects of stimulants deserve special attention. Many remain concerned regarding whether medications for neuropsychiatric conditions might interfere with creativity (Carson, 2011). However, our examination of this topic, as well as a recent review focusing on creativity and ADHD (Hoogman et al., 2020), show that while there is mixed evidence for the benefit for stimulants for creativity, there is not substantial evidence that it interferes with creativity. However, recreational use for performance enhancement for productivity and creativity is an important topic that deserves further consideration going forward, handled with considerable ethical equipoise.

Finally, further imaging studies may be helpful in disentangling these effects, including studies examining activation of regions associated with these systems, anatomical correlates, and potentially tracer imaging studies examining the functionality of these systems more directly. Very few papers have examined both convergent and divergent task performance with imaging paradigms. Recent reviews have highlighted the complex networks involved with creativity, with the highly overlapping networks associated with curiosity, involving the frontal, hippocampal, and subcortical areas, and areas involved in dopaminergic reward systems (Ivancovsky et al., 2024), and recent work has shown that frontopolar neurons are engaged during exploration (Nougaret et al., 2024), also known to be related to dopaminergic activity, and activity of the frontopolar region and its connectivity are critical in creativity task performance (Green et al., 2015). Also, the subthalamic medial zona incerta has been implicated in novelty-seeking behavior, with connectivity to the hippocampus, frontal cortex, and dopaminergic-associated networks (Ahmadlou et al., 2021; Londei et al., 2024). Systematic efforts at understanding how neurotransmitter systems interact with these brain regions during creativity is critical.

Disentangling these effects may have significant clinical implications. It would be informative for patients with conditions impacting the dopaminergic system (Parkinson’s and Tourette’s, for example), conditions impacting the noradrenergic system (anxiety disorders, for example), and conditions impacting both systems (ADHD, for example), to know the impact of their condition on performance, as well as the effect of their treatments on such performance. Finally, while the bulk of the literature on neurotransmitter systems has focused on NE and DA, there is evidence for the involvement of other systems, such as the serotonergic and cholinergic systems, which deserves attention in future studies, both as independent aspects and for how they interact with other systems (Beversdorf, 2018).

## Figures and Tables

**Table 1:** Effects on convergent and divergent performance associated with dopaminergic (DA) system manipulations and proxies; x = not assessed. Directionality of effect was deferred for anatomical and genetic measures, due to the complexity of causality on directionality. Tasks utilized are underlined. *Articles added after the described search are indicated in italics.* (tx = treatment; BIST = Berlin Intelligence Structure Test; RAT = Remote Associates Test; PCT = Picture Concepts Task; TOL = Tower of London; AUT = Alternate Uses Test; PLMT = Pattern/Line Meanings Task; TTCT = Torrence Test of Creative Thinking; FCIT = Finke’s Creative Invention Task; ASK = Analyse Schlussfolgernden und Kreativen Denkens; ATTA = Abbreviated Torrance Test for Adults; IPS = Insight Problem Solving; CRA = Compound Remote Associates; rCAB = Runco Creative Assessment Battery; TCIA = Test of Creative Imagery Abilities, PD = Parkinson’s disease, Conv = convergent, Div = divergent, ↑ = increased, ↓ = decreased, No eff = no effect).

Studies with DA Targeted	Conv	Div
**Studies with DA DRUGS**		
DA agonist healthy anagrams Smyth and Beversdorf (2007)	↓	x
DA blocker healthy BIST—possible uses, specific traits, symbol completion, object design subtasks Käckenmester et al. (2019)	x	Effect ^a^
DA drug cocaine RAT, PCT, TOL, AUT, PLMT Hutten et al. (2019)	↓ ^b^	↑↓ ^b^
DA agonist healthy and PD option generation task Ang et al. (2018)	x	↑
**Studies with DA DISEASES**		
Task performance Parkinson’s Guilford AUT, RAT Heldmann et al. (2024)	No eff	↑
Task perf. Tourette’s and healthy RAT, lines test from TTCT, FCIT, AUT, verbal puzzles (IPS task) Colautti et al. (2023)	↑ ^c^	No eff
Task perf. Tourette’s and Parkinson’s ASK test Zanaboni Dina et al. (2017)	x	↑ ^d^
Task perf. Parkinson’s ATTA Canesi et al. (2016)	x	No eff
Task perf. Parkinson’s AUT Varrone et al. (2015)	x	↓
**Studies with DA DISEASES PLUS DRUGS**		
DA replacement tx Parkinson’s AUT, RAT, Rebus puzzles Salvi et al. (2021)	No eff	No eff
DA tx Parkinson’s RAT, Tel Aviv Creativity Test, Novel Metaphors Faust-Socher et al. (2014)	x	↑ ^e^
**Studies with DA related tasks**		
Eyeblink rate and performance AUT Agnoli et al. (2023)	x	↑
Expect reward and performance CRA Cristofori et al. (2018)	↑	x
Eyeblink rate and performance AUT Chermahini and Hommel (2012)	x	Effect ^f^
Eyeblink rate and performance AUT, RAT Chermahini and Hommel (2010)	↓	↑
**Studies with DA genes**		
Perform with DA (DRD2 and COMT) genes AUT Chong et al. (2021)	x	Effect ^g^
Perform with DA (DRD2 and COMT) genes rCAB-figural divergent thinking Si et al. (2020)	x	Effect ^h^
Perform with DA gene DRD2 verbal puzzles, matchstick-style problems Zhang and Zhang (2016)	Effect	x
Perform with DA (DAT and COMT) genes ATTA Zabelina et al. (2016)	Effect ^i^	x
Perform with DA DRD2 Taq1 TTCT Takeuchi et al. (2015b)	x	Effect ^j^
Perform with DRD4 genes S-A creativity test Takeuchi et al. (2015a)	x	Effect
Perform with DA (DRD2 and COMT) genes verbal and figural divergent thinking tests from rCAB Zhang et al. (2014)	x	Effect ^i^
Perform with DRD4 genes AUT, TTCT (circles sub-scale) Mayseless et al. (2013)	x	Effect
Perform with DA gene DRD2 “inventiveness” battery of the BIST Reuter et al. (2006)	x	Effect
**Studies with DA imaging correlates**		
FC DA reg (pallid thal putamen) TTCT-figural Gao et al. (2021)	x	Effect
Microstructure R cortocistr path AUT, RAT, TCIA Rahmani et al. (2020)	Effect	Effect
fMRI active DA reg during DA task AUT, RAT Aberg et al. (2017)	Effect	Effect
fMRI activ DA assoc reg meta-Anal Various divergent tasks Wu et al. (2015)	x	Effect
Volume of DA assoc regions AUT, instances task Jauk et al. (2015)	x	Effect
Diffusivity in DA assoc regions S-A creativity test Takeuchi et al. (2015a)	x	Effect
DA D2 density thal PET inventiveness battery from the BIST de Manzano et al. (2010)	x	Effect
Volume of DA assoc ROIs S-A creativity test Takeuchi et al. (2010)	x	Effect
**Studies with NE targeted via electrical stimulation**		
Deep brain stim Parkinson’s ATTA Drago et al. (2009)	x	Effect

^a^ DA blocker increased divergence only with those with openness to experience. ^b^ Cocaine increased visual but decreased verbal divergence, convergence lower on only one figural task. ^c^ Effect related to impact of Tourette’s. ^d^ Tourette’s performance better than Parkinson’s. ^e^ With drug performed better than controls. ^f^ Inverted U relationship. ^g^ Effect of DA genes only when interacting with oxytocin. ^h^ Effect of DA genes only when interacting with parenting style. ^i^ Interaction between genes needed for effect. ^j^ Effect of gene requires interactions with motivational state, female gender, and emotional control.

**Table 2:** Effects on convergent and divergent performance associated with noradrenergic (NE) system manipulations and proxies. x = not assessed. Directionality of effect was deferred for anatomical and genetic measures, due to the complexity of causality on directionality. Tasks utilized are underlined. *Articles added after the described search are indicated in italics.* (RAT = Remote Associates Test; AUT = Alternate Uses Test; CRA = Compound Remote Associates, Conv = convergent, Div = divergent, ↑ = increased, ↓ = decreased, No eff = no effect).

Studies with NE Targeted	Conv	Div
**Studies with NE DRUGS**		
Propranolol (NE antag) in rodent Rodent Remote Problem Solving Maze Hecht et al. (2014)	↑	x
Propranolol (NE antag) healthy anagrams Silver et al. (2004)	↑	x
Propranolol (NE antag) healthy anagrams Beversdorf et al. (2002)	↑ ^a^	x
Propranolol (NE antag) healthy anagrams, Matchstick test Beversdorf et al. (1999)	↑ ^b^	x
*Propranolol (NE antag) healthy anagrams, CRA* Campbell et al. (2008)	*↑* ^c^	*x*
*Clonidine (α-2 agonist) healthy anagrams, CRA* Choi et al. (2006)	No eff	x
*Propranolol (NE antag) cocaine withdrawal anagrams* Kelley et al. (2007)	*↑*	*x*
*Propranolol (NE antag) autism anagrams* Zamzow et al. (2017)	*↑*	*x*
*Propranolol (NE antag) autism anagrams* Beversdorf et al. (2008)	*↑*	*x*
**Studies with NE targeted via stress**		
Stress monitoring Sympathetic Syst AUT, RAT Guo et al. (2024)	No eff	↑
*Stress impairs RAT perform* Martindale and Greenough (1975)	*↑*	*x*
*Stress impairs anagrams* Beversdorf et al. (2018)	*↑* ^d^	*x*
*Auditory stress impairs CRA* Hillier et al. (2006)	*↑*	*x*
*Stress impairs CRA* Renner and Beversdorf (2010)	*↑*	*x*
*Cold pressor and anagram & CRA* Ishizuka et al. (2007)	*No eff*	*x*
*Stress effect on CRA* Nair et al. (2020)	*↑* ^e^	*x*
**Stress with NE targeted via stress plus NE DRUGS**		
*Propranolol (NE antag) healthy, stress anagrams, CRA* Alexander et al. (2007)	↑	x
**Studies with NE targeted via sleep phase**		
Low NE assoc with REM sleep RAT Cai et al. (2009)	↑	x
*Low NE assoc with sleep phase anagrams* Walker et al. (2002)	↑	x
**Studies with NE targeted task**		
*Body position impacts anagrams* Lipnicki and Byrne (2005)	↑	x
**Studies with NE targeted via electrical stimulation**		
Vagal nerve stim anagrams, Abbreviated Torrance Test for Adults Ghacibeh et al. (2006)	Effect	Effect

^a^ Effect found in contrast with peripheral-only beta-adrenergic antagonist nadolol. ^b^ Effect found in contrast with adrenergic agonist ephedrine. ^c^ Effect found only for greatest task difficulty or individuals struggling with task. ^d^ Effect of stress found on those with stress-susceptible serotonin transporter polymorphism. ^e^ Effect of stress tended to be greater with stress-susceptible serotonin transporter polymorphism, with related differences on effects on functional connectivity with fMRI.

**Table 3:** Effects on convergent and divergent performance associated with manipulations and proxies of both DA and NE. x = not assessed. Directionality of effect was deferred for anatomical and genetic measures, due to the complexity of causality on directionality. Tasks utilized are underlined. *Articles added after the described search are indicated in italics.* (RAT = Remote Associates Test; AUT = Alternate Uses Test; TTCT = Torrence Test of Creative Thinking; ATTA = Abbreviated Torrance Test for Adults; CRA = Compound Remote Associates; rCAB = Runco Creative Assessment Battery; ANT = Alternate Names Task; GEFT = Group Embedded Figures Task, Conv = convergent, Div = divergent, ↑ = increased, ↓ = decreased, No eff = no effect).

Studies with NE and DA Targeted	Conv	Div
**Studies with NE and DA DRUGS**		
Stimulants ADHD CRA, TTCT-verbal McBride et al. (2021)	No eff	↑
Stimulants healthy AUT, RAT, ANT Baas et al. (2020)	No eff	No eff
Stimulants healthy AUT, RAT, ANT Sayalı et al. (2023)	x	↑↓ ^a^
Precursor (tyrosine) healthy RAT, AUT Colzato et al. (2015)	↑	No eff
*Stimulants ADHD AUT, Instances Test* Solanto and Wender (1989)	*x*	*↑*
*Stimulants ADHD TTCT-Figural* Funk et al. (1993)	*x*	*No eff*
*Stimulants ADHD AUT, Instances Test* Douglas et al. (1995)	*x*	*↑*
*Stimulants ADHD Test of Divergent Thinking* Swartwood et al. (2003)	*x*	*↓* ^d^
*Stimulants healthy RAT, GEFT, AUT, Drawing task from ATTA* Farah et al. (2009)	*↑* ^f^	*No eff*
*Stimulants ADHD TTCT-Figural* González-Carpio Hernández and Serrano Selva (2016)	*x*	*↓*
*Stimulants ADHD (between subject) RAT, AUT, Pasta task* Boot et al. (2017)	*No eff*	*No eff*
*Stimulants healthy AUT, TTCT-Figural* Gvirts et al. (2017)	*x*	*↑↓* ^e^
**Studies with NE and DA GENES**		
COMT genes rCAB-figural divergent thinking Si et al. (2020)	x	Effect ^b^
COMT genes ATTA Zabelina et al. (2016)	Effect	x
COMT genes verbal and figural divergent thinking tests from rCAB Zhang et al. (2014)	x	Effect ^c^

^a^ Depends on baseline DA synthesis capacity. ^b^ Relation depends on parenting style. ^c^ Effect found in interaction with DA genes. ^d^ Only lower performance on one aspect (elaboration subscale). ^e^ Increased in those with lower novelty seeking but decreased in those with higher novelty seeking. ^f^ Embedded figures task enhanced only for lower performing and RAT impaired only for higher performing individuals (RAT).

## Supplementary Materials

The following supporting information can be downloaded at: https://www.mdpi.com/article/10.3390/bs15091185/s1, Figure S1: PRISMA 2020 flow diagram reviews which included searches of databases.
