# Supplementary material for: Dopaminergic and Noradrenergic Contributions to Divergent and Convergent Creativity Task Performance, a Systematic Review
Source: Behav Sci (Basel). 2025 Aug 30;15(9):1185. doi: 10.3390/bs15091185 (PMC12466433; doi:10.3390/bs15091185)
Supplement: Supplementary file 1 [file behavsci-15-01185-s001.zip › behavsci-3770168-supplementary.pdf]

Supplemental Figure S1 PRISMA 2020 flow diagram reviews which included searches of databases

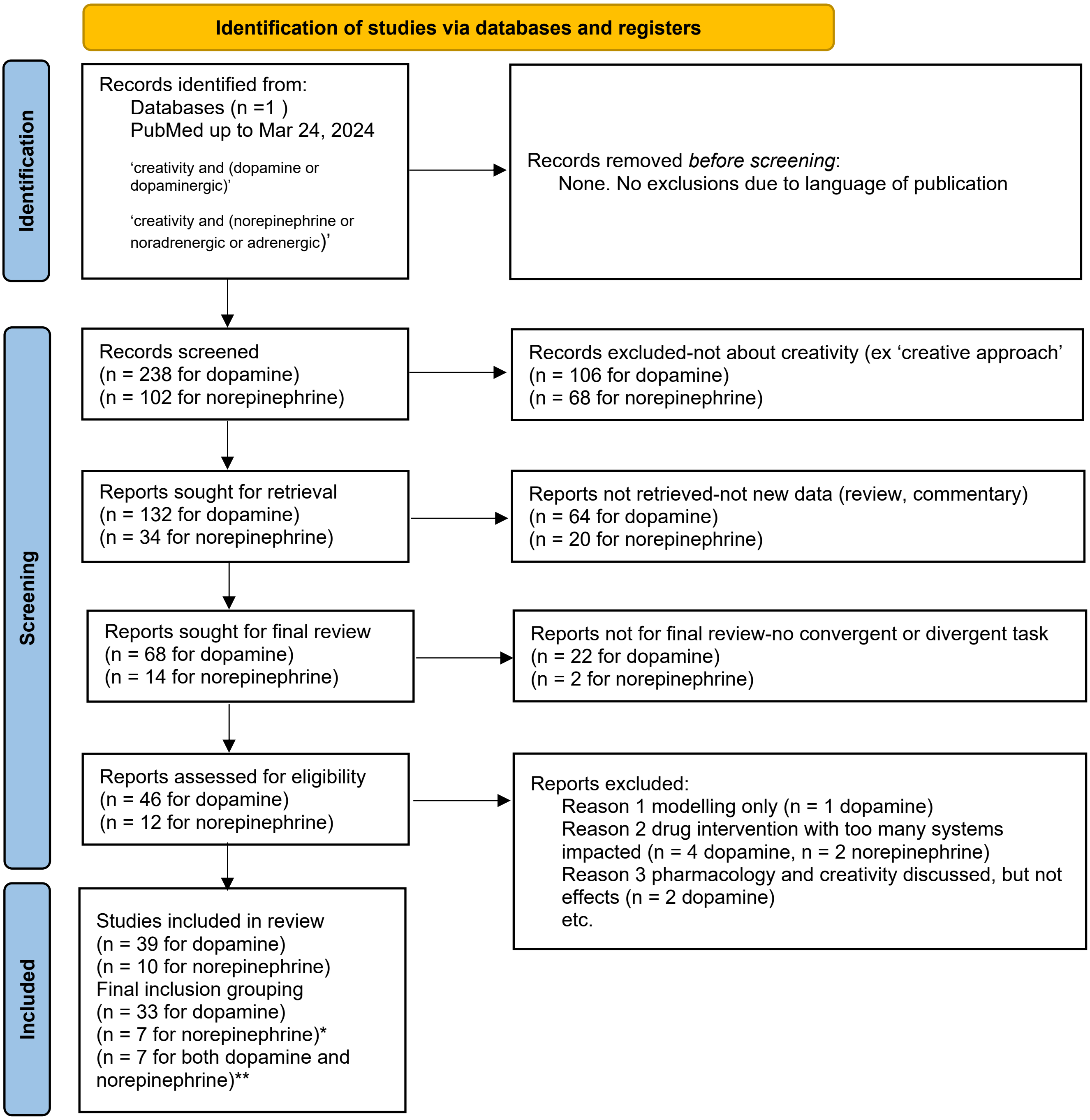

* = 12 additional papers known to the author added not captured by search strategy, as 'cognitive flexibility' was used rather than 'creativity' as the term
** = 8 additional papers known to the author added not captured by search strategy as the pharmacological systems were not highlighted in the discussion of the effects of stimulants
